# Cardiac Stem Cell Therapy for Cardiac Repair

**DOI:** 10.1007/s11936-014-0324-3

**Published:** 2014-06-07

**Authors:** Kyung U. Hong, Roberto Bolli

**Affiliations:** 1 Institute of Molecular Cardiology, Division of Cardiovascular Medicine, Department of Medicine, University of Louisville, Louisville, KY 40202 USA; 2 Diabetes and Obesity Center, Division of Cardiovascular Medicine, Department of Medicine, University of Louisville, Louisville, KY 40202 USA

**Keywords:** Stem cell therapy, Cardiac repair, Ischemic cardiomyopathy, Myocardial infarction

## Abstract

The discovery of adult cardiac stem cells (CSCs) and their potential to restore functional cardiac tissue has fueled unprecedented interest in recent years. Indeed, stem-cell–based therapies have the potential to transform the treatment and prognosis of heart failure, for they have the potential to eliminate the underlying cause of the disease by reconstituting the damaged heart with functional cardiac cells. Over the last decade, several independent laboratories have demonstrated the utility of c-kit+/Lin- resident CSCs in alleviating left ventricular dysfunction and remodeling in animal models of acute and chronic myocardial infarction. Recently, the first clinical trial of autologous CSCs for treatment of heart failure resulting from ischemic heart disease (Stem Cell Infusion in Patients with Ischemic cardiOmyopathy [SCIPIO]) has been conducted, and the interim results are quite promising. In this phase I trial, no adverse effects attributable to the CSC treatment have been noted, and CSC-treated patients showed a significant improvement in ejection fraction at 1 year (+13.7 absolute units versus baseline), accompanied by a 30.2 % reduction in infarct size. Moreover, the CSC-induced enhancement in cardiac structure and function was associated with a significant improvement in the New York Heart Association (NYHA) functional class and in the quality of life, as measured by the Minnesota Living with Heart failure Questionnaire. These results are exciting and warrant larger, phase II studies. However, CSC therapy for cardiac repair is still in its infancy, and many hurdles need to be overcome to further enhance the therapeutic efficacy of CSCs.

## Introduction

In the United States alone, myocardial infarction (MI) affects a total of 7.6 million people, and every year the associated mortality exceeds 125,000 people [1]. Despite significant advances in the management of MI, current therapeutic approaches do not address the central problem of the loss of functional myocardium, and there remains a large population that will develop heart failure (HF), leading to premature death. Currently, left ventricular assist devices (LVADs) and heart transplantation remain the only options available to patients with end-stage HF. For this reason, the ongoing research on cardiac stem cells (CSCs) and their innate ability to restore differentiated, functional cardiac tissue have kindled unprecedented interest in recent years. Indeed, stem cell–based therapies have the potential to transform the treatment and prognosis of HF, for they aim at eliminating the underlying cause of the disease by reconstituting lost myocardium with a new network of functional cardiac cells.

Although initial attempts to use autologous CSCs to treat ischemic cardiomyopathy are quite promising, the mechanisms that control their cellular behaviors and fate (e.g., survival, growth and differentiation) are poorly understood. Enhanced understanding of the basic biology of this unique cardiac cell population is essential to learn and predict their behaviors, address current problems, and ultimately, fully harness and augment their inherent reparative activity for the CSC-based therapies. For this reason, we will devote the first part of the present review to discussing some of the fundamental, yet unresolved, issues surrounding the origin and nature of the endogenous c-kit+/Lin- CSCs. In the second part, we will describe the history and current status of CSC therapy for ischemic cardiomyopathy. Finally, we will describe some of the problems of current CSC therapy and discuss the implications of different approaches that are currently being tested within the CSC research community, including our own research group, for tackling the existing issues. Of note, a comprehensive review on cell therapy for heart failure has been recently published elsewhere [2]. Here, we shall focus our discussion on the resident c-kit+/Lin- CSCs originally discovered by Anversa and colleagues [3].

## Identity, origin, and fate of c-kit+/Lin- CSCs

### Evidence for existence of cardiac stem/progenitor cells

In spite of the numerous conflicting reports concerning the regenerative capacity of myocardium, the majority of researchers agree that there is a profound difference in the regenerative capacity of skeletal and cardiac muscles. In the mammalian heart, it is generally accepted that adult cardiomyocytes do not possess the ability to proliferate. Hence, they had long been classified as *elementi perenni* or cells of static cell populations [4, 5]. Based on this view, the participation of a pool of structurally undifferentiated myoblasts or “myogenic stem cells” in cardiac myogenesis had been hypothesized many decades ago [6, 7]. Only recently, however, has the identity of such a heart-specific stem cell pool emerged.

In 2007, Hsieh and colleagues provided strong evidence that stem cells renew adult mammalian cardiomyocytes [8]. Although this study was preceded by the discovery and characterization of different cardiac progenitor cell populations, including c-kit+ cardiac stem cells (CSCs), it provided the first clear evidence for the presence of an undifferentiated cell population that directly contributes to myocardial renewal upon injury. The authors utilized transgenic mice in which the cardiomyocyte-specific *Myh6* (α-myosin heavy chain) promoter drives the expression of tamoxifen-inducible Cre recombinase (“αMHC-MerCreMer” mice) [9]. These mice were crossed with another transgenic mouse line that carries a reporter transgene which expresses β-galactosidase by default, yet begins to express enhanced green fluorescent protein (EGFP) upon Cre-mediated excision of the β-gal sequences [10]. By selectively activating Cre in cardiomyocytes, the authors introduced permanent EGFP expression in most cardiomyocytes in the adult mouse heart, whereas non-cardiomyocytes, including undifferentiated progenitors, remained β-gal-positive and EGFP-negative. Interestingly, when MI was experimentally induced in these hearts, there was a significant decrease in the percentage of EGFP-positive cardiomyocytes with a concomitant increase in the percentage of β-gal-positive cardiomyocytes. This implies that a β-gal-positive/EGFP-negative (i.e., non-myocyte) progenitor cell population contributed to the cardiomyocyte renewal following MI-induced injury to the myocardium. Although no specific progenitor cell population responsible for the cardiomyocyte renewal was identified in this study, it provided strong evidence for the existence of progenitor cell population(s) capable of contributing to de novo cardiomyogenesis following injury. However, it should be also noted that in normal aging mouse hearts (up to 1 year), the percentage of EGFP-positive cardiomyocytes did not change, suggesting that there is no significant contribution by the progenitor cells to the maintenance of myocardium under steady state conditions. The study raised a couple of important questions with regard to the mechanisms of maintenance and renewal of myocardium. *What is the identity of the progenitor/stem cell population? Are they necessary and sufficient for the maintenance and renewal of adult heart?*

### Identity of resident cardiac stem/progenitor cells

So far, the best-characterized and well-studied cardiac stem cell (CSC) population is the c-kit+/Lin- cells. c-kit+/Lin- cells with properties of CSC were first described in the rat heart by Beltrami et al. in 2003 [3]. The authors initially found rare c-kit+/Lin- cells present in the rat myocardium. Based on the presence of a stem cell marker, c-kit [11, 12] and the absence of pan-leukocyte marker CD45 and endothelial/hematopoietic progenitor marker CD34, it was proposed that c-kit+/Lin- cells represent a unique stem/progenitor population resident in the heart [3]. These cardiac cells exhibited a high nucleus to cytoplasm ratio and were relatively rare (one per 10,000 myocytes on average), yet were observed at a higher density in the atria and the ventricular apex. They were usually present in small clusters, most of which contained cells at early stages of cardiac myogenic differentiation, as demonstrated by the expression of the transcription factors Gata4, Nkx2.5, and Mef2, together with low levels of sarcomeric proteins in the cytoplasm. When isolated and grown in culture, c-kit+/Lin- cells exhibited extensive self-renewal capacity and clonogenicity, being able to proliferate for more than 19 months without reaching growth arrest or senescence. In addition, when induced to differentiate in vitro, they gave rise to cardiomyocytes, smooth muscle, and endothelial cells, although in vitro differentiated cells were morphologically and functionally immature. Furthermore, when injected into an ischemic heart, they reconstituted differentiated myocardium with new vessels and myocytes. Since then, c-kit+/Lin- CSCs have been described in multiple mammalian species, including human [13–15].

The c-kit+/Lin- cardiac cell population likely represents a heterogeneous cell population that contains lineage-committed progenitor cells along with true stem cells. Anversa and colleagues reported that c-kit+/Lin- cardiac cells included subpopulations that appeared to be more committed along the cardiac differentiation (i.e., lineage restricted) based on co-expression of c-kit and transcription factors of cardiovascular lineages, such as GATA-4, MEF2C, GATA-6, and Ets1 [14, 15]. Accordingly, a hierarchy among c-kit+ cardiac cells was proposed, in which c-kit+ cardiac cells are further categorized into different subpopulations of progenitor cells with variable growth and differentiation characteristics [16]. Recently, the same group also identified a coronary vascular progenitor cell (VPC) population in the human heart [17]. VPCs were found in specialized niches in epicardial coronary arteries, arterioles, and capillaries, and characterized by co-expression of c-kit and KDR/VEGFR2, an early marker of angioblast precursors [18]. They also expressed low levels of the endothelial cell adhesion molecule CD31 and the smooth muscle cell marker TGF-β1 receptor, while being negative for hematopoietic markers (e.g., CD45 and CD34) and α-sarcomeric actin. Upon differentiation, VPCs preferentially gave rise to the cells of vascular lineages (i.e., endothelial and smooth muscle cells) both in vitro and in vivo [17], indicating that they are more committed to the vascular lineages. Collectively, the c-kit+/Lin- cardiac cell population may represent several different lineage-committed progenitor cell populations along with true stem cells. Such finding emphasizes the need for more rigorous characterization of the c-kit+/Lin- cardiac cell population, which is used in both preclinical and clinical settings. It is uncertain if the proportions of different progenitors remain relatively constant or vary among individuals, especially depending on the disease state or other physiological variables. It will be of great interest to study how the changes in cellular composition of the c-kit+/Lin- cardiac cell population translate into differences in proliferative and differentiation potentials, and ultimately, in the therapeutic efficacy of patient-specific c-kit+ CSC populations.

Interestingly, several other resident cardiac progenitor/stem cell populations capable of contributing to tissue maintenance and repair have also been reported in adult mammalian hearts. These progenitor/stem cell populations were initially identified based on their expression of a common stem cell antigen *Sca-1* [19, 20], or on their ability to efflux a fluorescent dye, Hoechst 33342 (“Side Population” [SP]) [21–23], or to form spherical bodies (“cardiospheres”) under a specific culture condition [24–26]. Additionally, epicardium-derived progenitor cells that express *Wt1*, an embryonic epicardiac gene, have been shown to exhibit characteristics of cardiac stem cells [27, 28]. However, their true identities are still controversial, and it is not clear how these different stem/progenitor cell populations are related to one another. *Does each truly represent stem/progenitor population in the heart? Are they distinct, mutually exclusive populations? Or do they represent subpopulations of progenitor cells at different stages of commitment?* Dey and colleagues attempted to address these important questions by comparing the global gene expression profiles of three different cardiac stem/progenitor cell populations in age-matched C57BL/6 mice: c-kit+/CD45- CSCs (c-kit+), Sca-1+/CD31-/CD45- (Sca-1+) cells, and “Side Population” (SP) cells [29]. The microarray gene expression patterns of these cardiac populations were also compared to those of bone marrow (BM)-derived c-kit+ cells and BM-derived mesenchymal stem cells (MSCs) in the study. c-kit+ cardiac cells represented at most 3 % of the non-myocyte small cells, and yet 1–2 % co-expressed c-kit and the pan-leukocyte marker, CD45. Sca-1+ cardiac cells accounted for approximately 8–10 % of the unfractionated cardiac cell population, while SP cells represented only 0.9 %. Based on comparative analysis of their gene expression profiles and hierarchical clustering, Sca-1+ cells were closest to cardiomyocytes, followed by SP cells, and c-kit+ cells were distinct from the other two populations and cardiomyocytes. Compared to c-kit+ cells, Sca-1+ and SP cells showed relatively higher expression of myocyte-specific transcription factors, contractile proteins, ion channels, and calcium-binding proteins, suggesting that Sca-1+ and SP cells are more committed along the myogenic differentiation pathway. Interestingly, several hematopoietic cell-specific genes were upregulated in c-kit+ cells with respect to Sca-1+ and SP cells. It is unclear if this is simply due to a small number of c-kit+/CD45+ double positive cells still present in the population or if this reflects their tissue origin (see more discussions below). However, cardiac c-kit+ cells were clearly distinct from their BM counterparts with respect to their gene expression profiles. Of note, cardiosphere-derived cells were found to be more closely related to BM-derived MSCs [29], suggesting that they may represent heart-specific MSCs. In summary, this study indicated that cardiac c-kit+/CD45- cells (i.e., c-kit+ CSCs) are not only distinct from their BM-derived counterparts, but also represent the most primitive progenitor population in the mouse heart [29], further supporting the stem cell role of cardiac c-kit+/CD45- cells and their utility as a therapeutic engine.

### Origin of c-kit+/Lin- CSCs

The developmental origin, as well as the origin of adult c-kit+/Lin- CSCs have been controversial topics. As mentioned above, the initial characterization of adult c-kit+/Lin- CSCs revealed that they are negative for CD45 and CD34, suggesting that they are not of hematopoietic origin [3]. Also, the presence of specialized CSC niches within the heart argues for their cardiac origin. The CSC niches contain clusters of undifferentiated c-kit+ CSCs and early cardiovascular lineage-committed cells (i.e., c-kit and GATA4, MEF2C, or Ets1 double-positive cells) [15]. Moreover, within the CSC niche, CSCs and early lineage-committed cells are connected to neighboring myocytes and fibroblasts via gap and adherens junctions [15]. Taken together, these observations strongly suggest that CSCs not only reside stably in the heart, but are also specifically committed to give rise to multiple cardiac cell types. Recently, Ferreira-Martins et al. provided evidence that c-kit+ CSCs originate from the developing heart itself by using transgenic mice that express GFP under the control of the c-kit promoter (c-kit-GFP). The authors utilized two-photon microscopy to directly monitor in situ the temporal and spatial changes in the GFP+ (i.e., c-kit+) putative CSC population in the heart of developing c-kit-GFP transgenic embryos [30]. Starting at E6.5, a cluster of c-kit+ cells was detected in the cardiogenic mesoderm, and by E8.0, c-kit+ cells in the heart tube were observed migrating towards the apex. Importantly, the authors reported that migration of extra-cardiac GFP+ (i.e., c-kit+) into the heart was not observed during the window of observation, arguing for the cardiac origin of c-kit+ CSCs. However, it should be noted that rigorous and continuous monitoring of GFP+ cells in a developing embryo is technically challenging, and thus the possibility that a small number of extra-cardiac (e.g., hematopoietic) stem cells contributing to the cardiac c-kit+ cell population and to the developing myocardium could not be completely ruled out by the study.

In contrast, several studies have suggested that c-kit+ cardiac cells may not represent a stable resident cell population but have an extra-cardiac origin, such as BM. This view is, in part, supported by the intriguing observations that cardiomyocytes of non-cardiac origin are detected in patients who have received gender-mismatched heart [31, 32], BM [33, 34], and peripheral blood stem cell [33] transplants. Fazel and colleagues [35] have shown that c-kit+ cells are mobilized from the BM and recruited to the heart following MI, and serve cardio-protective functions. Interestingly, the majority of them downregulated and no longer expressed CD45, a pan-hematopoietic cell surface marker. Similarly, others have shown that hematopoietic stem cells are recruited into the damaged myocardium and contribute to cardiac repair [36, 37]. These studies raised an interesting possibility that the c-kit+ CSCs may not originate from the heart, but from the BM [38]. A recent study tested this idea by creating chimeric mice whose BM had been reconstituted, with one constitutively expressing GFP [39•]. Using these BM chimeric mice, one can easily identify BM-derived cells in different organs based on the GFP expression. The chimeric mice were subjected to isoproterenol-induced heart injury, and the presence of BM-derived GFP+ cells in the regenerated heart was examined at 28 days of recovery. The authors were unable to find any GFP+ cardiomyocytes in the restored myocardium, clearly demonstrating that BM-derived cells do not directly contribute to myocyte renewal. Interestingly, they also noted that a small fraction of c-kit+/CD45- cardiac cells was GFP+ (i.e., of BM origin) in both control and isoproterenol-treated hearts [39•]. It is not clear, however, if these BM-derived c-kit+/CD45- cardiac cells eventually acquire the role of resident CSCs and contribute to the maintenance and renewal of myocardium.

It appears that a genetic fate mapping study is needed to resolve the issues surrounding the developmental as well as adult origin(s) of resident c-kit+ CSCs. Such studies would involve introducing permanent reporter gene expression (e.g., lacZ or GFP), specifically in cardiac mesoderm or early cardiac progenitor cells in the developing embryo, and tracking the reporter gene expression to the cardiac c-kit+/CD45- cell population in the adult heart.

### Contribution of c-kit+/Lin- CSCs to myocardial maintenance and renewal

Numerous studies have demonstrated that c-kit+/Lin- CSCs isolated and expanded in culture exhibit all the properties of bona fide stem cells, and when injected into the injured myocardium, they are capable of restoring (to a variable extent) the cardiac structure and function in various animal models, as well as in patients suffering from ischemic cardiomyopathy [3, 13–15, 40••, 41•, 42]. Results of recent animal/pre-clinical studies are summarized in Table 1. Moreover, CSC dysfunction has been implicated in development and progression of cardiac pathologies associated with type I diabetes and anthracycline toxicity [47–49]. However, the precise role of *endogenous* c-kit+ CSCs in maintenance and renewal of the heart has remained poorly understood. *Do c-kit+ CSCs truly contribute to tissue maintenance and renewal under physiological and pathological conditions? Do they possess multipotent differentiation potential in their physiological milieu? Is their plasticity real or just a cell culture artifact? Why does heart still lack sufficient regenerative capacity despite the presence of these resident CSCs?*

**Table 1:** Recent animal/pre-clinical studies of c-kit+ resident cardiac stem/progenitor cell therapy in heart failure

Study	Host	Type of heart failure	Time of cell therapy	Dose and route of administration	Follow-up period after cell therapy	Results
Rota et al. [43]	Rat	Permanent coronary occlusion	20 d after MI	40,000 cells Intramyocardial	2 wk	• ↑ LVEF • Attenuated matrix remodeling • ↓ Fibrosis • Cardiac regeneration & neo-vascularization • Improved hemodynamic parameters • ↓ LV remodeling
Tang et al. [41•]	Rat	Ischemia/reperfusion injury	30 d after MI	40,000 cells Intracoronary	35 d	• Attenuated matrix remodeling • ↓ Fibrosis • ↓ LV remodeling • Cardiac regeneration
Bolli et al. [44•]	Pig	Ischemia/reperfusion injury	90 d after MI	500,000 cells Intracoronary	31 d	• ↑ LVEF • Improved hemodynamic parameters • ↓ Fibrosis • ↓ LV remodeling • Cardiac regeneration and angiogenesis
Williams et al. [45]	Pig (immune-suppressed)	Ischemia/reperfusion injury	14 d after MI	Human CSCs (1 million cells) and/or human MSCs (200 million cells) Intramyocardial	4 wk	• ↑ LVEF • ↓ Infarct size • Attenuated matrix remodeling • ↑ LV chamber compliance & contractility • Attenuated remodeling • hCSC + hMSC treatment group superior over the rest
Mohsin et al. [46]	Mouse (SCID)	Permanent coronary occlusion	0 d after MI	Human CPCs (100,000 cells) or Pim-1-expressing CPCs; intramyocardial	20 wk	• ↑ LVEF • Improved hemodynamic parameters • ↓ Fibrosis • Enhancement of myocyte formation & neo-vascularization • Pim-1 hCPCs group superior over the rest

Recently, Ellison and colleagues [39•] employed an elegant method to tackle some of the aforementioned questions. The authors used a reversible, isoproterenol heart injury model [50] and a genetic fate mapping approach [51] to determine if c-kit+ CSCs are indeed able to renew the cardiomyocytes lost by myocardial injury. The normal mouse heart was administered (via local delivery) a lentivirus encoding Cre-recombinase under the regulation of the murine c-kit promoter (c-kit-Cre) to introduce a traceable genetic tag specifically into c-kit-expressing cardiac cells. The mice used for this experiment carried a reporter transgene (i.e., enhanced yellow fluorescent protein; EYFP), which is irreversibly activated upon Cre-mediated recombination [52]. Once EYFP expression is introduced to c-kit+ cardiac cells, it can be traced down to all of their differentiated progenies. Lentivirus-mediated delivery of c-kit-Cre introduced EYFP expression in 38 ± 5 % of total and 65 ± 7 % of apex-confined c-kit+ CSCs. The mouse hearts with EYFP-labeled c-kit+ CSCs were then subjected to isoproterenol-induced cardiac injury. After 4 weeks, only 0.06 ± 0.02 % of total cardiomyocytes were EYFP+ in control mice, whereas 9.5 ± 2 % of total cardiomyocytes (approximately 59 % of newly formed, BrdU-labeled cardiomyocytes) were EYFP+ in the isoproterenol-treated group, indicating that some of the newly generated cardiomyocytes indeed originated from the c-kit+ CSCs. Although this method does not rule out the participation of other cardiac stem/progenitor populations [20, 28, 53] or pre-existing cardiomyocytes [54] to the myocardial repair, the study was the first to demonstrate the c-kit+ CSCs contribute to the cardiomyocyte renewal by direct differentiation in vivo. Unfortunately, it was not mentioned whether or not EYFP label was also transmitted down to other cell types in the heart, including smooth muscle and endothelial cells. *Are c-kit+ CSCs unable to differentiate into vascular cell types in vivo or could this be injury-dependent? Do they also contribute to normal turnover of the myocardium?* It would be of great interest if the same genetic fate mapping strategy was applied to investigate the role of c-kit+ CSCs in a more clinically relevant model of cardiac injury, such as MI induced by ischemia-reperfusion. Of note, as we were preparing this manuscript, Molkentin and colleagues developed such fate mapping mouse model for c-kit+ cells and reported a finding that questions the myogenic potential of c-kit+ cardiac cells [55].

As mentioned earlier, administration of an exogenous pool of c-kit+ CSCs can either reverse or significantly attenuate various cardiac pathologies [40••, 41•, 42, 47, 49]. However, studies involving transplantation of CSCs expanded ex vivo do not address the issue of whether or not they are *necessary* for myocardial maintenance and renewal. If one were able to control the fate of c-kit+ CSCs in vivo, it would be possible to address this issue. For instance, selective depletion of c-kit+ CSCs in a temporally controlled manner followed by histological as well as functional assessments of the CSC-depleted heart would tell us whether or not c-kit+ CSCs are indeed indispensible for myocardial maintenance and repair. *Does CSC-depleted heart function normally or deteriorate with normal aging or following injury?* Other important questions, perhaps of greater clinical significance, also remain. *How can we stimulate or activate the endogenous pool of c-kit+ CSCs? Does it provide meaningful therapeutic benefits?* Currently, it is technically challenging to address these important and fundamental questions. It would require better understanding of their unique molecular phenotype to devise methods and genetic tools that allow cell-specific manipulation of this unique cardiac population in vivo.

## c-kit+ CSC-based therapy for ischemic cardiomyopathy

### c-kit+ CSC-based therapy in pre-clinical animal models

During the last decade, several independent laboratories have well demonstrated the ability of human and rodent CSCs to promote cardiac regeneration and alleviate MI-induced left ventricular (LV) dysfunction and remodeling in various animal models of acute MI [14, 42, 56–58]. However, the therapeutic efficacy of intra-coronarily infused CSCs in the setting of an old MI (induced by a temporary coronary occlusion followed by reperfusion) would be very relevant to the contemporary clinical settings. To this end, Tang et al. tested the reparative activity of CSCs in mitigating cardiac dysfunction and remodeling using a rat chronic MI model [41•]. One month after coronary occlusion/reperfusion injury, rats received an intracoronary infusion of GFP-labeled CSCs. At 5 weeks post-transplantation, compared to the vehicle controls, CSC-treated hearts exhibited improvements in both LV structure and function, as demonstrated by greater viable myocardium in the risk region, less fibrosis in the non-infarcted region, and improved ejection fraction (EF). However, despite the structural and functional improvements, GFP+ donor cells were rarely found in the recipient hearts, and those co-expressing cardiomyocyte markers (e.g., α-sarcomeric actin) did not exhibit the typical morphology and sarcomeric structure of mature cardiomyocytes (see more discussions below) [41•]. In order to further confirm this finding in a large, clinically relevant species, a similar study was performed in pigs [44•]. Three months after coronary occlusion-reperfusion injury, autologous CSCs were infused into the infarct-related artery using a balloon catheter. At one month post-infusion, pigs treated with CSCs exhibited a significant increase in LV function, indicated by an increase in EF and systolic thickening fraction in the infarcted LV wall, as well as a decrease in LV end-diastolic pressure [44•]. The efficacy of CSCs in the setting of chronic ischemic cardiomyopathy was noteworthy, especially when considering the potentially hostile environment provided by the old scar and the lack of robust stimulatory signals that can home and activate the transplanted stem cells. These encouraging results from the animal studies formed the basis for the first clinical trial of c-kit+ CSCs, Cardiac Stem Cell Infusion in Patients with Ischemic Cardiomyopathy (SCIPIO) [40••, 59••].

### SCIPIO trial

SCIPIO was a phase I (randomized, open-label) trial of autologous c-kit+ CSCs for the treatment of ischemic HF [40••, 59••]. The study design and results are summarized in Table 2. Patients with left ventricular ejection fraction (LVEF) of ≤40 % who underwent coronary artery bypass grafting (CABG) were the target population. Patient-specific c-kit+/Lin- CSCs were isolated from the right atrial appendage harvested during surgery and further expanded in culture. Approximately 4 months after CABG, 20 patients were administered with 10^6^ autologous CSCs by intracoronary infusion; 13 control patients did not receive any treatment. The interim results are quite encouraging and promising. In CSC-treated patients, magnetic resonance imaging (MRI) analysis showed a marked increase in LVEF (from 27.5 ± 1.6 % to 35.1 ± 2.4 %) at 4 months after the infusion, whereas LVEF did not change in the control subjects [59••]. The beneficial effects of CSCs became even more pronounced at 1 year (from 27.5 ± 1.6 % to 41.2 ± 4.5 %) [59••]. In addition, there was a profound reduction in infarct size at 4 months (−6.9 ± 1.5 g [−22.7 %]) and at 1 year (−9.8 ± 3.5 g [−30.2 %]) after CSC infusion, which was accompanied by a significant increase in LV viable mass [40••, 59••]. Moreover, these CSC-induced enhancements in cardiac function were associated with a significant improvement in the New York Heart Association (NYHA) functional class and in the quality of life (measured by the Minnesota Living with Heart Failure Questionnaire). Importantly, no adverse effects attributable to the CSC treatment were noted. The results of the SCIPIO trial indicate that that intracoronary delivery of autologous c-kit+ CSCs is not only safe, but also leads to a significant and clinical improvement in patients with ischemic cardiomyopathy, which is supported by the substantial and continuous improvement in LV systolic function and reduction in infarct size in the treated group.

**Table 2:** Clinical study of c-kit+ resident cardiac stem/progenitor cell therapy in heart failure

Study	Design	Number of patients	Method of delivery	Cell dose	Follow-up period	Results	Reported side effects of therapy
Bolli et al. (SCIPIO) [40••]	Open-label, randomized, controlled study	Control (*n* = 7); cell-treated (*n* = 16)	Intracoronary	1 million cells	4 mo and 12 mo	↑ LVEF ↓ Infarct size ↓ NYHA class	No major complications

The degree of cell engraftment and mechanisms of the CSC-mediated clinical outcomes are currently unknown. Considering that animal studies have shown that c-kit+ CSCs suffer poor survival and retention following transplantation [60], we speculate that the transplanted cells trigger native regenerative response (e.g., recruitment of endogenous stem cells) via secretion of paracrine factors.

## Challenges in CSC therapy

### Survival and retention of c-kit+ CSCs following transplantation

A number of studies have reported that stem cells of various sources suffer low viability, and only few persist several weeks following transplantation into the injured myocardium [60–67]. Similarly, one of the problems with the current therapy with c-kit+ CSCs is poor survival and retention of the injected cells in the heart [41•, 42, 60]. It is thought that myocardial ischemia/reperfusion and infarction create a hostile environment for stem cells, because of the presence of inflammatory cells and cytokines/mediators, lack of extracellular matrix and supporting cells, and poor supply of oxygen and nutrients, all of which conspire to promote death of the grafted cells [68, 69]. For this reason, increasing survival and retention of the transplanted CSCs in the heart currently constitutes one of the major challenges in the field of CSC therapy.

Troubleshooting this issue would require close and accurate monitoring of the number, distribution, and fate of the transplanted donor cells, and correlation of these variables with changes in functional parameters. Our research group has recently developed a novel quantitative PCR (qPCR)-based method to quantify the *absolute* numbers of male mouse CSCs remaining in the female recipient heart at a given time point [60]. The method involves adding cells of another genotype (e.g., human cells) to each tissue sample as an internal standard prior to genomic DNA isolation. Since the ratio of the internal standard DNA (i.e., human DNA) to the male mouse donor DNA should remain constant regardless of the efficiency of DNA isolation, the amount of human DNA in the sample can be used to calculate the total, original amount of male donor DNA present in the entire tissue. The amount of DNA of a particular genotype is acquired by qPCR and converted to the number of cells by dividing it by the amount of genomic DNA present per cell (e.g., 6.26 pg DNA per diploid human cell). Another unique feature of the assay is that it targets a novel, male-specific, multiple-copy gene named *Rbmy* [70, 71], which increases the sensitivity of detection of male DNA by several folds over a traditional male marker, *Sry* [60].

In our previous study [60], we measured the absolute numbers of male CSCs remaining in the recipient heart following intramyocardial or intracoronary delivery into the female mouse heart. When intramyocardial injection of 10^5^ CSCs were made (at 2 days after ischemia-reperfusion), only 43 % of the injected cells were found at 5 min after injection. This immediate and massive cell loss is likely due to leakage of cells through transepicardial puncture holes. Greater than 75 % of CSCs present at 5 min were lost in the ensuing 24 h, and only 7.6 % of the CSCs present at 5 min were found at 7 days. By 35 days, only 2.8 % of the cells present at 5 min (i.e., 1,224 ± 257 cells/heart; 1.2 ± 0.2 % of total cells injected) were detected in the recipient heart. Following intracoronary infusion of the same number of CSCs (i.e., 10^5^), > 60 % of the injected CSCs were already lost by 5 min, suggesting that the majority of cells failed to be retained and were washed off almost immediately due to the coronary blood flow. Moreover, more than 85 % of the cells at 5 min were lost in the next 24 h. Only 3.5 % and 2.4 % (i.e., 987 ± 211 cells/heart; 1.0 ± 0.2 % of total cells injected) of the cells present at 5 min were found at 7 days and 35 days, respectively. In addition to the heart, presence of the intracoronarily delivered donor cells was detected in lungs and kidneys of the recipient mice, yet they were absent in livers and spleens (Hong et al., manuscript submitted). Although the number of donor cells in the heart declined slightly faster following intracoronary infusion (versus intramyocardial injection), the percentage of transplanted cells remaining after 35 days was similar between the two different delivery methods, reaching approximately 1 % (i.e., ~1,000 cells out of 100,000). These quantitative studies clearly demonstrate that the transplanted CSCs suffer low survival and/or retention in the recipient heart, regardless of the delivery methods, and strongly support the notion that the poor survival and/or retention of the donor CSCs is indeed a major factor limiting the efficacy of the current CSC therapy.

Several studies have tested different strategies to overcome the problem of poor survival or retention of the cells in the hostile environment of the infarcted heart. For example, Mohsin and colleagues tested the effect of ex vivo gene delivery of a pro-survival gene, Pim-1 kinase on survival/engraftment and reparative potential of human CSCs using a mouse model of ischemic cardiomyopathy [46]. The same group had previously identified Pim-1 as a kinase responsible for cardioprotection downstream of pro-survival Akt signaling [72, 73]. Human CSCs engineered to overexpress Pim-1 were superior over the control cells in terms of cellular engraftment and differentiation. Bioluminescence imaging analysis of luciferase-expressing donor cells [74] showed that the presence of Pim-1 CSCs persisted up to 8 weeks post-transplantation, whereas control cells became undetectable by 1 week. Moreover, the persistence of cells in the recipient heart was accompanied by improvement in vasculature and reduction in infarct size, which were coupled with increased hemodynamic performance at 20 weeks post-transplantation [46]. Interestingly, a subsequent report by the same group showed that the increases in proliferation and telomere lengths observed in Pim-1-expressing CSCs are only transient, suggesting that Pim-1 overexpression does not lead to immortalization or oncogenic transformation of CSCs [75]. The results are promising and indicate that ex vivo delivery of a pro-survival gene prior to transplantation may serve a viable option in overcoming current limitations in the field.

Such strategy also has proven fruitful in terms of promoting survival and therapeutic efficacy of other stem cell sources, including MSCs [61, 76, 77]. One of the first attempts to improve post-transplantation donor MSC survival was reported by Mangi and colleagues [78]. They genetically engineered rat MSCs ex vivo using retroviral transduction to overexpress the pro-survival gene *Akt1* [79, 80]. At 24 h following intramyocardial injection of MSCs into the rat MI heart, they observed a greater number of donor cells and a lower rate of apoptosis with MSCs overexpressing Akt (Akt-MSC) compared to the control GFP-expressing MSCs [78]. Subsequently, transplantation of MSCs into the ischemic heart significantly reduced intramyocardial inflammation, fibrosis and myocyte hypertrophy, and normalized cardiac function. The study reported that Akt-MSCs restored fourfold greater myocardial volume compared to the control MSCs. Another study tested the utility of stably introducing heme oxygenase-1 (HO-1) expression in MSCs [61]. HO-1 is known to exert a potent anti-apoptotic, anti-oxidant and cytoprotective activity in an ischemic environment [81, 82]. When they injected HO-1 MSCs into acute MI heart, their survival was fivefold greater than that of the control MSC group (expressing lacZ) at 7 days of implantation, and this was consistent with a significantly reduced rate of apoptosis among HO-1 MSCs [61]. Importantly, HO-1 MSCs were also superior over the control cells in attenuating LV remodeling and enhancing the functional recovery of injured hearts [61]. Similarly, the pro-survival or cardio-protective effects of various candidate genes, including GATA4 [76], heat shock protein 27 [83], miRNA-1 [84], and protein kinase G1α [85], have been recently exploited to promote survival and efficacy of MSCs in the context of hypoxia-reoxygenation injury.

Pharmacologic activation of innate cytoprotective mechanisms seems to be another attractive option to enhance the survival and engraftment of CSCs. In a recent study, Cai et al. showed that treatment of human c-kit+ CSCs with cobalt protoporphyrin (CoPP), a well known HO-1 inducer [86], promoted cell survival after increased oxidative stress in vitro [87]. The cytoprotective effects of CoPP were dependent on the upregulation of HO-1, cyclooxygenase-2 (COX-2), and nuclear factor-like 2 (NRF2). Interestingly, preconditioning CSCs with CoPP also led to a global increase in release of a variety of cytokines, and the conditioned medium from cells pretreated with CoPP conferred naive CSCs remarkable resistance to apoptosis, demonstrating that cytokines released by preconditioned cells also play a major role in the pro-survival effects of CoPP [87]. Another study by Zafir and colleagues examined the role of protein O-GlcNAcylation (β-O-linkage of N-acetylglucosamine, O-GlcNAc), a universal cytoprotective pathway [88] in the protection of mouse CSCs against hypoxia-reoxygenation injury [89]. Protein O-GlcNAcylation was significantly elevated in post-hypoxic CSCs upon reoxygenation. Reduction in the O-GlcNAc signal via pharmacological inhibition sensitized CSCs to post-hypoxic injury, whereas increasing O-GlcNAc levels enhanced cell survival. The study has an important implication that pharmacologic augmentation of protein O-GlcNAc levels can be effective in priming CSCs for survival within the unfavorable environment of the infarcted myocardium. Likewise, preconditioning of cells prior to transplantation or co-treatment with different agents has been shown to be a successful strategy for reinforcing viability and therapeutic efficacy of the donor MSCs [90–94]. To this end, our group has recently discovered that activation of c-kit-mediated signaling enhances viability of CSCs under stress conditions in vitro. The preliminary data indicate that activation of c-kit by its ligand, stem cell factor, not only prolongs the survival of c-kit+ CSCs under serum deprivation, but also stimulates their migration robustly in vitro (Vajravelu and Hong, unpublished observations). It would be of interest to see if activation of c-kit receptor on CSCs can enhance viability of the donor cells, as well as their ability to home into the infarcted myocardium.

Preconditioning or priming the stem cells with pharmacological agents prior to autologous transfer is likely to be clinically viable, for it is relatively easy to implement and does not involve direct genetic manipulation of the cells. Undoubtedly, identification of important cytoprotective mechanisms within CSCs and potential pro-survival candidate genes will be critical in developing novel strategies for augmenting post-transplantation survival and engraftment of donor CSCs.

### Lack of robust, direct differentiation of transplanted CSCs

Although a variety of stem cell populations, including c-kit+ CSCs, have been shown to play regenerative and/or protective functions following transplantation in animal models of acute and chronic myocardial infarction, it has been widely accepted that therapeutic benefits of stem cell therapy are mainly contributed by paracrine factors secreted by the donor cells, rather than by direct differentiation of transplanted stem cells into cardiac cell types [69, 95–98]. Consistent with this notion, our group has also observed that most of the transplanted c-kit+ CSCs do not give rise to mature cardiomyocytes, despite that a number of the transplanted cells begin to express markers of cardiomyocytes [41•, 42]. Even after 35 days post-transplantation, nearly all of the cardiomyogenic progenies (based on α-sarcomeric actin expression) of the donor cells were small and failed to exhibit striations. The reason for this is not clear, but may be due to either alteration of the cellular properties during ex vivo expansion or lack of proper molecular and cellular cues at the site of transplantation. Regardless of the cause, there is a disconnection between functional benefits of CSC therapy and the lack of robust differentiation of transplanted cells. Based on this, one can argue that the contribution of direct cardiovascular differentiation of the donor stem cells to the salutary effects is negligible.

However, previous studies on transplantation of non-cardiac stem cells suggests that direct cardiac differentiation of the donor stem cells is essential for the functional benefits they provide. Yoon and colleagues investigated the contribution of cardiovascular differentiation of BM-derived mononuclear cells (BMMNCs) to the renewal of myocardium following acute MI by using a clever system in which the differentiated progenies of the donor cells can be selectively ablated in a temporally controlled manner [99]. They engineered the donor cells to express a vector that encoded a prodrug-activated suicide gene (*herpes simplex virus thymidine kinase*; *HSVtk*) under the control of endothelium (eNOS)-, smooth muscle (SM22α)-, or cardiomyocyte (α-MHC)-specific promoters, which permitted selective depletion of the individual cardiac lineage acquired by the donor cells via administration of a prodrug, ganciclovir. When each donor cell-derived lineage was ablated two weeks after transplantation of the engineered BMMNCs, they found that depletion of endothelium-committed or smooth-muscle–committed cells, but not cardiomyocyte-committed cells, induced a significant decline in EF [99]. This demonstrated that vascular differentiation is one of the essential mechanisms by which BMMNCs contribute to the functional recovery of the injured heart.

Another study by Behfar et al. also suggested that cardiogenic potential of the donor BM-derived MSC population dictates the functional outcome of stem cell therapy [100•]. While the majority of patient-derived MSC failed to elicit significant improvements in EF, stem cells from few individuals harbored a spontaneous capacity to improve cardiac performance in an animal model of chronic ischemic cardiomyopathy. Interestingly, the stem cells exhibiting the reparative activity expressed relatively high basal levels of early (e.g., NKX-2.5, TBX5, and MESP1) and late (e.g., MEF2C) cardiac transcription factors, compared to the non-effective MSC counterparts. Based on this observation, the authors formulated a “cardiogenic cocktail” (containing TGF-β1, BMP-4, activin A, retinoic acid, IGF-1, FGF-2, α-thrombin, and IL-6) to stimulate cardiogenic potential/differentiation of naïve MSCs prior to transplantation. This strategy not only potentiated expression of cardiac transcription factors across different patient MSC populations, but also enhanced therapeutic efficacy of MSCs against chronic ischemic cardiomyopathy in mice [100•]. The study provides an interesting implication that enhancing differentiation potential or properties of donor stem cells is feasible, and is an effective strategy to further improve the efficacy of stem cell therapy. Along the same lines, our research group has been exploring the idea of “priming” CSCs prior to transplantation to facilitate their differentiation. We have been experimenting with the approach of “forward reprogramming” human CSCs by introducing individual or combination of different cardiac transcription factors, including GATA4, NKX-2.5, MEF2C, TBX5, and BAF60C. Our results in vitro are promising that overexpression of GATA4 potentiates expression of marker genes associated with multiple cardiovascular lineages in human CSCs (Hong and Al-Maqtari et al., manuscript in revision). It remains to be seen whether or not such strategy can lead to robust and full differentiation of human CSCs following transplantation, and thereby enhance their regenerative activity in the infarcted heart.

## Concluding remarks

The results of the first clinical trial (SCIPIO) of autologous c-kit+/Lin- CSCs for treatment of ischemic cardiomyopathy are promising and clearly warrant larger studies. However, CSC therapy for cardiac repair is still in its infancy, and much more work will be required to better understand the basic biology of these cells and to enhance their reparative activity. We believe that this work will be crucial to fully harness the therapeutic potential of CSCs.
